# DSM-5 and autism spectrum disorders (ASDs): an opportunity for identifying ASD subtypes

**DOI:** 10.1186/2040-2392-4-12

**Published:** 2013-05-15

**Authors:** Rebecca Grzadzinski, Marisela Huerta, Catherine Lord

**Affiliations:** 1 Center for Autism and the Developing Brain, Weill Cornell Medical College and New York Presbyterian Hospital/Westchester Division, NY, USA; 2 Teachers College, Columbia University, NY, USA

## Abstract

The heterogeneous clinical presentations of individuals with autism spectrum disorders (ASDs) poses a significant challenge for sample characterization and limits the interpretability and replicability of research studies. The *Diagnostic and Statistical Manual of Mental Disorders*, 5th edition (DSM-5) diagnostic criteria for ASD, with its dimensional approach, may be a useful framework to increase the homogeneity of research samples. In this review, we summarize the revisions to the diagnostic criteria for ASD, briefly highlight the literature supporting these changes, and illustrate how DSM-5 can improve sample characterization and provide opportunities for researchers to identify possible subtypes within ASD.

## Review

Over the past several decades, researchers have attempted to categorize the heterogeneity in autism spectrum disorders (ASDs) [1-9]. This effort has been largely unsuccessful because distinct, empirically defined subgroups have yet to be reliably identified. The *Diagnostic and Statistical Manual of Mental Disorders*, 4^th^ edition (DSM-IV) used a multi-categorical system of diagnosing pervasive developmental disorders (PDDs), which included autistic disorder, Asperger’s disorder, pervasive developmental disorder not otherwise specified, childhood disintegrative disorder, and Rett’s disorder, that created challenges to this effort. A number of studies have reported limited reliability in how DSM-IV subtypes are assigned [10,11], with similar core symptom presentations across the categorical diagnoses [12-16] and poor predictive ability of later outcome based on these subtypes [17,18]. Consequently, the fifth edition of the DSM (DSM-5) replaces the multi-categorical system with a single diagnostic dimension: ASD.

Although concerns have been raised about the validity and diagnostic sensitivity of the proposed DSM-5 criteria [19], a number of studies have emerged in support of the conceptual validity of the new criteria [20]. Huerta *et al*. [21] found that over 90% of children with DSM-IV-defined PDDs were identified by DSM-5 criteria, and the specificity using the new diagnostic criteria was substantially improved compared with the DSM-IV criteria.

### Key changes in the DSM-5 ASD criteria

The change in DSM-5 that received the most media attention is the removal of the DSM-IV clinical subtypes. Less publicized were the content changes and the new symptom structure in DSM-5. Over the past two decades, an increase in access to large and diverse samples has given researchers the ability to determine that, in many cases, ASD symptoms are best represented in a two-domain model of social-communication deficits and restricted and repetitive interests/behaviors (RRB) [22-25], rather than by the DSM-IV triad of symptoms that models communication deficits separate from social impairments. In addition, although the criteria for DSM-IV Autistic Disorder required a delay in or complete lack of development in expressive language, this requirement has been eliminated in DSM-5 because research has shown that this characteristic is neither specific nor universal to individuals with ASD [26-30].

Changes within symptom domains have also been warranted. DSM-5 includes unusual sensory responses in the RRB domain to reflect research showing that these behaviors are prevalent in ASD [31-34] and are useful in distinguishing ASD from other disorders [35,36]. One feature of unusual communication, stereotyped language, has been reassigned to the RRB domain to reflect results from factor analytic studies [25]. Other DSM-IV symptoms have been retained in DSM-5, but their definition has been revised in order to increase specificity.

### Improved methods for subtyping?

The emphasis on specificity in the DSM-5 improves researchers’ ability to identify samples of interest. In addition, DSM-5 introduces a dimensional approach that allows researchers to capture variability within samples in two important ways. First, although DSM-5 requires that symptoms from both the RRB and social-communication domains are present, it allows individual variation in the quality and quantity of specific RRBs and social-communication deficits. Second, DSM-5 formally recognizes many features that are not specific to ASD by which researchers can qualify ASD diagnoses.

Some individuals with ASD have unique patterns of social-communication deficits and RRBs, suggesting a possible avenue by which ASD subgroups might be defined. For example, results from a longitudinal study suggested that RRB quantity at 2 years of age is inversely related to language skills at 9 years [37]. With respect to the quality of symptoms, there is some evidence to suggest that ‘insistence on sameness’ behaviors are distinct from other core ASD features and from symptoms of anxiety [3]. ‘Insistence on sameness’ behaviors also seem to be independent of autism symptom severity, age, and intelligence quotient (IQ), suggesting that it may be a useful qualitative characteristic by which to identify ASD subtypes [38].

Individuals with ASD also display substantial variation in the presentation of sensory abnormalities. The DSM-5 criteria describe any unusual sensory response to the environment, sensory reactivity, or unusual sensory interests, within one dimension. Research has begun to explore the possibility of distinct ASD subgroups based on specific patterns of sensory abnormalities [39,40]. Further explorations are required in the area of sensory abnormalities and in other dimensions of the RRB symptoms. Similarly, research has demonstrated the utility of subgrouping within ASD based on specific social-communication profiles [4]. Researchers should therefore consider both the quality and quantity of specific ASD symptoms across social-communication and RRBs, as these may provide useful constructs to define ASD subgroups. This approach may also help to address the broader conceptual question of whether (and which) core ASD diagnostic symptoms lie along continuums, and whether these symptoms are similarly continuous in non-ASD samples.

Beyond the variability in the presentation of core symptoms of ASD, DSM-5 recognizes that affected individuals also vary with respect to non-ASD symptoms, such as cognitive ability, expressive language ability, onset patterns, and comorbid psychopathology. These distinctions may provide additional means by which to identify subtypes within ASD. Below, we highlight the literature that illustrates how the dimensional approach of DSM-5 can result in opportunities for researchers to identify possible subtypes within ASD.

In terms of cognitive functioning, individuals with ASD display a wide range of abilities, from severe intellectual disability (ID) to superior intelligence. Studies examining ASD individuals with and without ID have found significant differences between groups in symptom severity and later outcomes [41,42]. Moreover, deficits in cognitive functioning may be more common in females [43-46], in individuals with known genetic abnormalities [47,48] and in individuals with dysmorphic features [49], suggesting the possible existence of subgroups with unique etiologies or risk factors. In addition, cognitive profiles based on the relative discrepancy between verbal and non-verbal skills in ASD have been identified [50,51]. However, clear ASD subtypes based on specific cognitive deficits, with or without ID, have yet to be clearly defined [1].

**Figure 1:**
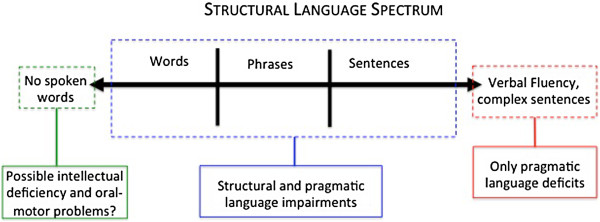
Illustration of the range of structural language impairment presentations in autism spectrum disorder.

As noted above, DSM-5 does not consider language delays to be part of the core symptoms of ASD because level of language ability is highly variable in ASD. That is, individuals with ASD differ in the degree to which they master structural aspects of language (such as syntax, morphology, and phonology) [52,53] ranging from individuals who never develop spoken language to individuals with intact structural language abilities (who speak in fluent, complex sentences), yet still have deficits in the pragmatic use of language (Figure 1). These outcomes have been associated with unique clinical presentations; for example, individuals who are non-verbal have more severe symptoms of ASD, ID [54], and oral-motor difficulties [55]. Researchers have also suggested that a subset of individuals with ASD may present with language deficits that are similar to the impairments of individuals with specific language impairment (SLI), such as limited production of expressive language and deficits in grammar and syntactical structure. However, whether these deficits in individuals with ASD are distinct from or are on a continuum of deficits seen in SLI without ASD requires further investigation [53].

Research exploring the utility of structural language deficits as neurobiological markers of subgroups has been promising. Neurobiological evidence suggests that individuals with both ASD and language impairment show similar patterns of structural brain abnormalities in the core language areas of the brain (for example, Wernicke’s and Broca’s areas) as those individuals with SLI without ASD, whereas individuals with ASD without language impairment do not show this pattern [56]. Along the same line, genetic variation in the gene CNTNAP2 (contactin-associated protein-like 2) has been implicated in ASD and SLI research separately, further supporting the potential of a subtype of ASD with comorbid SLI and unique underlying biological mechanisms [57]. A more recent study found that siblings of individuals with SLI and ASD traits were more likely than the general population to have a diagnosis of ASD, providing further evidence for possible combined genetic loadings of ASD and SLI [58]. This line of inquiry emphasizes the necessity for researchers to thoroughly consider the quality of language impairments in individuals with ASD, as clinically meaningful variability may extend beyond simple delay in language acquisition.

The recognition in DSM-5 of an individual’s onset pattern as a clinical specifier and the removal of the specific age of onset requirement should encourage researchers to carefully document these details for each case. Individuals with ASD differ widely in the timing and type of symptom onset. According to a recent meta-analysis of 85 studies, about 30% of individuals with ASD display regressive onset profiles, usually occurring around 18 months of age [59]. Four specific types of regression patterns have been suggested: regression in language skills, regression in language and social skills, regression in motor or adaptive functioning skills, or unspecified regression [59]. However, other studies have identified different patterns of regression [60-64] and some researchers suggest that regressions should be considered along a continuum [65]. The research is also mixed on whether certain patterns of regression are associated with specific outcomes. In some studies, regression-onset patterns have been associated with lower verbal ability, more severe social impairments [66,67], and possible higher rates of epilepsy [68,69], whereas other studies have found no differences between groups [61,70]. These mixed findings may be the result of variability in how regression is operationalized across studies [59] and the challenges that arise when parents confuse plateauing skills with regression [71,72]. Additional research is needed to understand whether different onset patterns represent subgroups of individuals with unique trajectories or biological underpinnings.

Individuals with ASD often, but not always, present with features consistent with additional non-ASD disorders, such as attention deficit/hyperactivity disorder (ADHD), anxiety disorders, or mood disorders [73]. In the case of individuals with ASD and comorbid ADHD, symptoms tend to include more social deficits and more general psychopathology than found in those individuals with ASD only [74]. Furthermore, there is evidence that individuals with ASD and symptoms of combined subtype ADHD display a profile of difficulties (such as increased oppositional behaviors) that is unique from those with ASD and inattentive subtype ADHD [75]. Results from community-based twin studies provide evidence of shared heritability in ASD and ADHD [76,77]; therefore, exploration into the overlapping and distinct phenotypic presentations of individuals with comorbid presentations (ASD with ADHD) versus presentations of ADHD or ASD singly may be of potential importance for understanding the biological markers of these disorders.

### What should researchers do?

**Figure 2:**
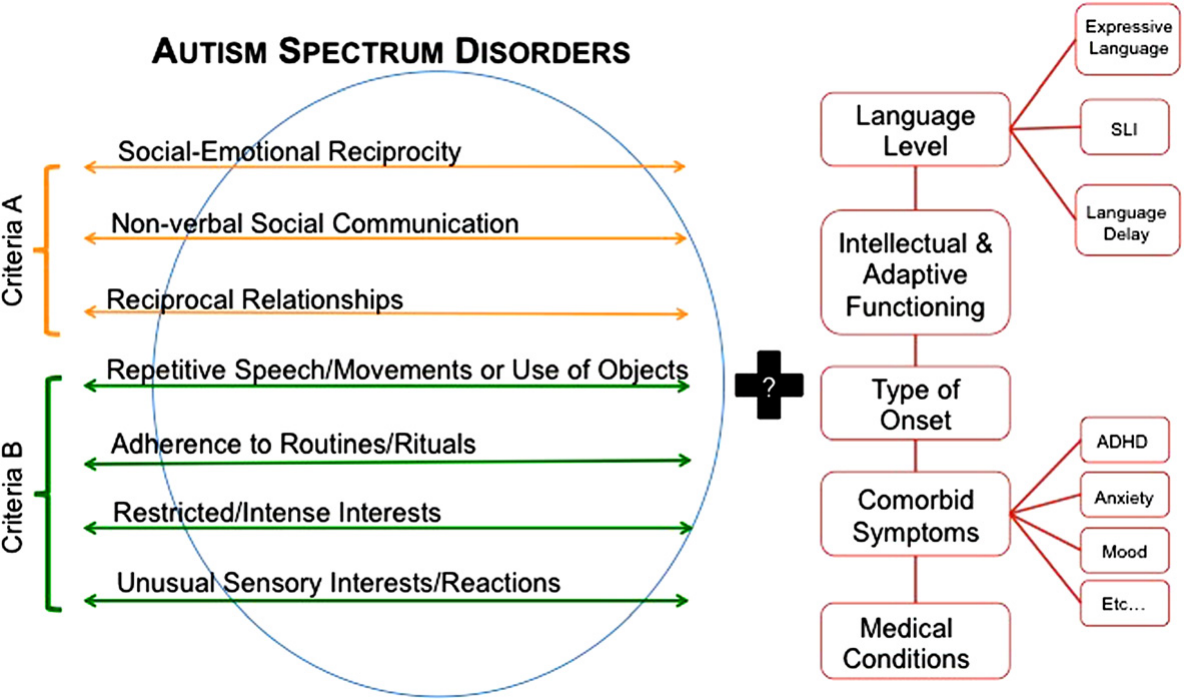
**Proposed Diagnostic and Statistical Manual of Mental Disorders, 5**^**th **^**edition (DSM-5) criteria and associated features to be considered when characterizing autism spectrum disorder (ASD) samples.**

In an effort to increase the specificity of the diagnostic criteria, DSM-5 identifies both core diagnostic symptoms and non-ASD-specific characteristics that vary within ASD populations. Taken together, these revisions encourage researchers to take a dimensional approach when studying the heterogeneous autism phenotype, similar to approaches that have been used in population samples [78]. Because of variations in samples (for example, age or IQ), no single study will be sufficient to accurately define ASD subgroups, but the emerging accumulation of research can begin to bolster understanding in this area. However, in order to advance in this line of inquiry, is it essential that researchers use adequate sample characterization. In an effort to provide guidance to researchers, Figure 2 illustrates the proposed DSM-5 criteria. In the next section, we expand on this list and highlight the associated features that researchers should consider when characterizing their ASD samples.

### ASD-specific symptoms

Qualitative and quantitative identification of ASD-specific symptoms requires case confirmation of research samples, using diagnostic measures that provide both qualitative and quantitative data on the core deficits in ASD.

### Cognitive and adaptive functioning

In addition to assessing whether or not a comorbid classification of ID is warranted, an individual’s abilities within cognitive domains should also be assessed (verbal and non-verbal abilities in samples with and without ID). Another key consideration may be patterns of discrepancy between cognitive and adaptive abilities, as some individuals with ASD have difficulty with daily living skills despite having adequate cognitive skills [79,80].

### Language skills

Language skills should be assessed and described, including the current level of receptive and expressive language skills and the quality of possible SLI.

### Pattern of onset

The onset pattern should be defined, including the presence or absence of early language or other skill regression. This includes thorough reports about specific skill acquisition, regression, or plateau, as well as reports about more global developmental delays.

### Comorbid symptoms

Identification of comorbid symptoms should include both those symptoms that are sufficient for a comorbid diagnosis and those symptoms that may not meet full criteria for a diagnosis but are relevant as a descriptor of an individual’s phenotype. In some cases, this will require that characterization of research samples rely on skilled clinicians in combination with valid measures that are capable of identifying comorbid psychopathology in individuals with ASD.

### Other medical conditions

Researchers should investigate whether an individual has other medical conditions including genetic conditions and abnormalities (for example, Fragile X, Rett’s disorder, Down syndrome), dysmorphology, and other general medical conditions (for example, diabetes, celiac disease).

### Other behaviors of concern

Researchers should seek to identify other behaviors of concern. DSM-5 allows for characterization of those individuals with increased behavioral problems, eating difficulties, and sleeping abnormalities.

## Conclusions

In sum, the dimensional approach of the DSM-5 is comprehensive in that it recognizes both core ASD symptoms and clinical features that are not specific to ASD. This approach to sample characterization will help researchers to increase the homogeneity of their research samples, thus enhancing the interpretability and replicability of their work. The ultimate goal is to identify subgroups within ASD that will be important for understanding the biological mechanisms, clinical outcomes, and treatment responses of individuals with ASD.

## Competing interests

CL receives royalties for the Autism Diagnostic Observation Schedule and the Autism Diagnostic Interview–Revised; all such royalties from clinics and projects in which she is involved are donated to charity.

## Authors’ contributions

RG, MH, and CL were involved in conception, review of research, interpretation, writing and revision of the article. All authors read and approved the final manuscript.
